# Functional Screening Identifies MicroRNA Regulators of Corin Activity and Atrial Natriuretic Peptide Biogenesis

**DOI:** 10.1128/MCB.00271-19

**Published:** 2019-11-12

**Authors:** Selvi Celik, Mardjaneh Karbalaei-Sadegh, Göran Rådegran, J. Gustav Smith, Olof Gidlöf

**Affiliations:** a Department of Cardiology, Clinical Sciences, Lund University, Lund, Sweden; b The Hemodynamic Lab, Section for Heart Failure and Valvular Heart Disease, Skane University Hospital, Lund, Sweden; c Department of Heart Failure and Valvular Heart Disease, Skane University Hospital, Lund, Sweden

**Keywords:** atrial natriuretic peptide, cardiomyocyte, corin, heart failure, microRNA

## Abstract

Atrial natriuretic peptide (ANP) represents an attractive therapeutic target in hypertension and heart failure. The biologically active form of ANP is produced by the cardiac serine protease corin, and modulation of its activity might therefore represent a novel approach for ANP augmentation. MicroRNAs (miRNAs) are pervasive regulators of gene expression, but their potential role in regulating corin activity has not been elucidated. Our aim was to systematically identify and characterize miRNA regulators of corin activity in human cardiomyocytes. An assay for measuring serine protease activity in human induced pluripotent stem cell (iPS)-derived cardiomyocytes was used to perform a comprehensive screening of miRNA family inhibitors (*n* = 42). miRNA 1-3p (miR-1-3p) was identified as a potent inhibitor of corin activity. The interaction between miR-1-3p and a specific target site in the *CORIN* 3′ untranslated region (3′ UTR) was confirmed through argonaute 2 (AGO2)-RNA immunoprecipitation and reporter assays. Inhibition of miR-1-3p resulted in upregulation of *CORIN* gene and protein expression, as well as a concomitant increase in extracellular ANP. Additionally, miR-1-3p was found to interact with and inhibit the expression of several transcriptional activators of ANP gene expression. In conclusion, we have identified a novel regulator of corin activity and ANP biogenesis in human cardiomyocytes that might be of potential future therapeutic utility.

## Abstract (precis)

Atrial natriuretic peptide (ANP) represents an attractive therapeutic target in hypertension and heart failure. The biologically active form of ANP is produced by the cardiac serine protease corin, and modulation of its activity might therefore represent a novel approach for ANP augmentation. MicroRNAs (miRNAs) are pervasive regulators of gene expression, but their potential role in regulating corin activity has not been elucidated.

## INTRODUCTION

Atrial natriuretic peptide (ANP) plays an essential role in the regulation of blood pressure and sodium homeostasis (1). ANP is produced and released by atrial myocytes in response to increased intravascular volume and exerts potent diuretic and vasodilatory effects through binding to natriuretic peptide receptor A (NPR-A) (2). Consequently, ANP plays an important role in maintaining cardiorenal homeostasis in hypertension and heart failure. Augmentation of the natriuretic peptide system (NPS) as a therapeutic strategy was recently consolidated in the PARADIGM-HF trial, which showed that increasing natriuretic peptide half-life in the circulation through inhibition of neprilysin (in combination with renin-angiotensin-aldosterone system blockade) had beneficial effects on hospitalization times and cardiovascular deaths in heart failure (HF) patients (3). However, novel and more targeted strategies for NPS augmentation could provide a more effective treatment with fewer side effects.

ANP is transcribed from the *NPPA* gene and translated into the precursor peptide pro-ANP which, upon release, is cleaved by the cardiac transmembrane serine protease corin to form the biologically active peptide. Inhibition of *CORIN* expression blocks pro-ANP processing *in vitro* (4), and genetic ablation of *CORIN* results in salt-sensitive hypertension and cardiac hypertrophy (5, 6). In humans, loss-of-function variants in the *CORIN* gene are associated with hypertension, cardiac hypertrophy, and reduced natriuretic peptide processing (7–10). Moreover, HF is characterized by high levels of unprocessed pro-ANP and there are numerous indications that corin activity is decreased in experimental models of HF (11–13). Therefore, corin is an interesting therapeutic target to achieve NP augmentation in HF and hypertension. However, regulation of corin expression and catalytic activity has not been extensively studied. With regard to the regulation of *CORIN* gene expression, Pan et al. showed that there are functional, conserved GATA4 motifs in the *CORIN* promoter (14) and Lee et al. proposed a mechanism whereby regulated inositol-requiring protein 1 (RIE1) promotes corin mRNA degradation in the context of HF (15).

MicroRNAs (miRNAs) are a class of short, noncoding RNAs with pervasive roles in tissue homeostasis and disease (16–19). Through base pairing with complementary sequences in the 3′ untranslated region (3′ UTR) of mRNAs, miRNAs mediate repression of specific genes through mRNA degradation or translational repression (20). The tissue-specific expression of many miRNAs, combined with the relative ease with which miRNAs can be modulated *in vivo*, has made these molecules attractive therapeutic targets across a wide range of disease states, including cardiovascular disease (21).

miRNAs have been implicated in the regulation of ANP biogenesis, but only through direct binding to *NPPA* mRNA (22, 23). In the present study, we have conducted an unbiased, large-scale functional screening of miRNAs that inhibit corin activity in human cardiomyocytes. Such miRNAs could ultimately constitute therapeutic targets for NP augmentation.

## RESULTS

### Adaptation and validation of an assay for measuring corin activity in human cardiomyocytes.

**FIG 1:**
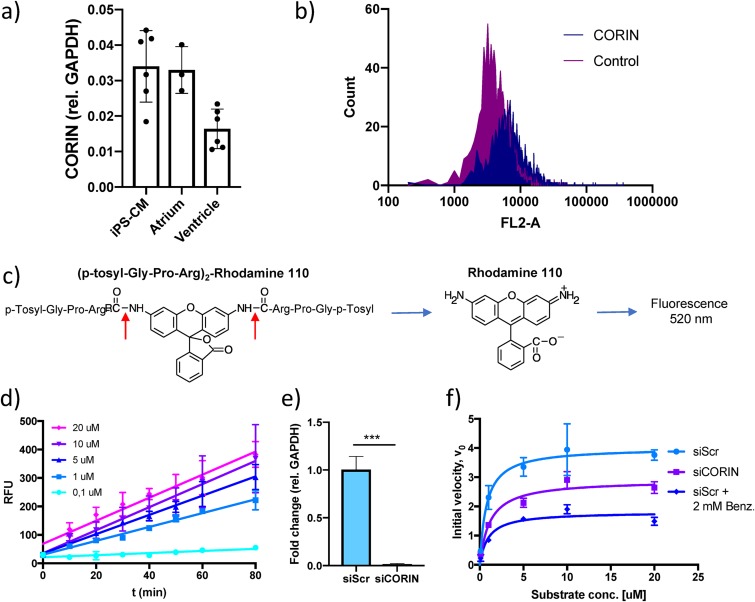
Results of an assay for measuring serine protease activity in human cardiomyocytes. (a) *CORIN* gene expression in induced pluripotent stem cell-derived cardiomyocytes (iPS-CM) and human cardiac tissue specimens of atrial and ventricular origin analyzed by qRT-PCR. Results are expressed relative to the expression of *GAPDH* (encoding glyceraldehyde-3-phosphate dehydrogenase) (*n* = 3 to 6). (b) Membrane expression of corin in iPS-CM analyzed by immunostaining and flow cytometry. Shown are representative histograms for negative-control cells stained only with secondary antibody and cells stained with a corin antibody. (c) Structure of the fluorogenic serine protease substrate used in the assay. The red arrows indicate the two serine protease cleavage sites of the substrate. (d) Reaction progress curves for a range of substrate concentrations. The increases in fluorescence were linear and proportional to substrate concentrations. Data are derived from three separate experiments. RLU, relative light units. (e) *CORIN* gene expression in iPS-CM transfected with siRNA for *CORIN* (siCORIN) or scrambled negative-control siRNA (siScr). Results are expressed relative to the results for *GAPDH* and are based on three separate experiments. ***, *P* < 0.001, using Student’s *t* test. (f) Michaelis-Menten saturation curves for cells transfected with scrambled control siRNA or *CORIN* siRNA and cells treated with 2 mM benzamidine. Data are derived from three separate experiments.

We applied the principle of the cardiac serine protease activity assay described by Chen et al. (13) to human induced pluripotent stem cell-derived cardiomyocytes (iPS-CM). First, the expression of *CORIN* in iPS-CM was assessed with reverse transcription-quantitative PCR (qRT-PCR) and found to be comparable to that in human atrial tissue (Fig. 1a). The presence of corin protein on the cell membrane of iPS-CM cells was confirmed using flow cytometry (Fig. 1b). Corin activity was measured by adding a fluorogenic bisamide substrate of rhodamine 110 containing two serine protease cleavage sites, (*p*-tosyl-Gly-Pro-Arg)_2_-Rho110, to cells in culture and monitoring the increase in fluorescence over time (Fig. 1c). The reaction progress curves were linear across a range of substrate concentrations (0.1 to 20 μM) (Fig. 1d), indicating that initial reaction velocity (*V*_0_) conditions were met. To test the validity of the assay, enzyme kinetics were compared between untreated control cells, cells treated with 2 mM serine protease inhibitor benzamidine, and cells transfected with *CORIN* small interfering RNA (siRNA). Successful knockdown of *CORIN* gene expression was confirmed with qRT-PCR (Fig. 1e). As expected, there was a 50% reduction in maximal reaction rate (*V*_max_) with the addition of benzamidine (from *V*_max_ of 4.01 [95% confidence interval {CI}, 3.3 to 4.98] in control cells to *V*_max_ of 1.81 [95% CI, 1.57 to 2.1] in benzamidine-treated cells). Knockdown of *CORIN* mRNA reduced *V*_max_ by 25% (to *V*_max_ of 2.0 [95% CI, 2.5 to 3.3]). Neither of the treatments significantly affected *K_m_* (Fig. 1f). Taken together, these results indicate that the assay is sensitive to cardiac serine protease activity in general and corin activity specifically.

### Screening of an miRNA family inhibitor library.

**FIG 2:**
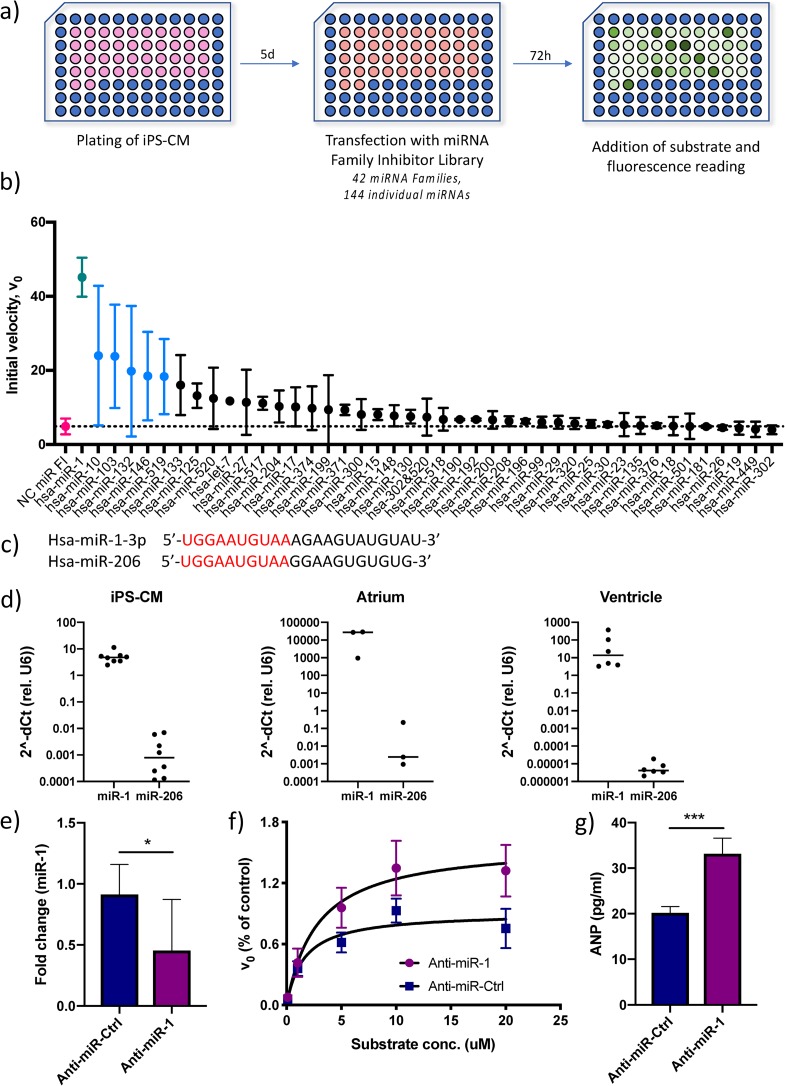
Identification of miR-1 as a regulator of cardiac serine protease activity and ANP biogenesis. (a) Overview of the screening procedure. (b) Initial reaction velocity (*V*_0_) for each miRNA family inhibitor ranked according to effect. Data for miRNA inhibitors that caused a statistically significant difference in *V*_0_ (*P* < 0.05 using Student’s *t* test) are shown in blue, and data for the only inhibitor whose results remained significant after adjusting for multiple comparisons (*q* < 0.05, using the false discovery rate approach), miR-1, are shown in green. Data for cells transfected with a negative-control (NC) miRNA family inhibitor are shown in red. Results are based on two separate screenings with two technical replicates each. (c) RNA sequences for the two miRNA-1 family members, miR-1-3p and miR-206, with the seed sequence shown in red. (d) Expression of miR-1-3p and miR-206 relative to that of U6 RNA in iPS-CM and human cardiac tissue specimens of atrial and ventricular origin, measured by qRT-PCR (*n* = 3 to 6). (e) Expression of miR-1 in iPS-CM transfected with LNA antisense inhibitor anti-miR-1 analyzed by qRT-PCR. Data are expressed relative to the results for U6 RNA and normalized to the mean value of the control cells (anti-miR-Ctrl). *, *P* < 0.05, using Student’s *t* test. Data are derived from three separate experiments. (f) Michaelis-Menten saturation curves for iPS-CM transfected with anti-miR-1 or scrambled control anti-miRNA. Results are based on three separate experiments. (g) Concentrations of ANP in the supernatants of iPS-CM transfected with anti-miR-1 or scrambled control anti-miRNA measured by enzyme-linked immunosorbent assay (ELISA). ***, *P* < 0.001, using Student’s *t* test. Data are derived from three separate experiments.

To identify miRNAs that affect corin activity in human cardiomyocytes, an miRNA inhibitor library was screened. To simplify the screening procedure, we utilized a library with inhibitors directed at miRNA families, rather than individual miRNAs. Members of an miRNA family share a “seed” sequence, i.e., the nucleotides at positions 2 to 8 of the miRNA which bind to target mRNAs. The library contained locked nucleic acid (LNA) antisense inhibitors for 42 miRNA families, covering a total of 144 individual miRNAs (see Table S1 in the supplemental material for detailed information about the miRNAs included). Human iPS-CM were transfected with the miRNA family inhibitor library 72 h prior to assaying serine protease activity. The initial reaction velocity (*V*_0_) at 20 μM substrate was used as the endpoint for screening (Fig. 2a). Six miRNA family inhibitors (corresponding to the miRNA 1 [miR-1], -10, -103, -132, -146, and -519 families) caused statistically significant increases in enzyme activity (*P* < 0.05), but only the result for the miR-1 inhibitor remained significant after adjusting for multiple comparisons (false discovery rate [*q*] < 0.05) (Fig. 2b). There were no examples of miRNA families where inhibition led to a decrease in corin activity. Inhibition of the miR-1 family caused a 10-fold increase in enzyme activity compared to the activity in the negative control. The miR-1 family consists of two members, miR-1-3p and miR-206 (Fig. 2c), whose expression is restricted almost exclusively to cardiac and skeletal muscle, respectively (24, 25). In fact, miR-1-3p was ranked as the single most tissue-specific miRNA in a recent study (25). Moreover, miR-1-3p is one of the most abundant miRNAs in cardiomyocytes, with reported roles in cardiac development (26) and disease (27–29). We confirmed that miR-1-3p was the predominant miR-1 family member in iPS-CM, as well as in human atrial and ventricular tissue (Fig. 2d). Given the cardiac expression profile of miR-1-3p, we chose to focus the remainder of the study on this miR-1 family member. Specific inhibition of miR-1-3p in iPS-CM using an LNA antisense oligonucleotide (anti-miR-1) caused a 60% reduction in miR-1-3p expression (Fig. 2e) and a corresponding 50% increase in *V*_max_ in the serine protease assay compared to the results for cells treated with a scrambled control anti-miR oligonucleotide (Fig. 2e). Moreover, miR-1 inhibition caused a significant increase in extracellular ANP from iPS-derived cardiomyocytes, adding further evidence that miR-1-3p directly affects corin activity (*P* < 0.001) (Fig. 2g).

### AGO2-RIP identifies corin as a direct target of miR-1.

**FIG 3:**
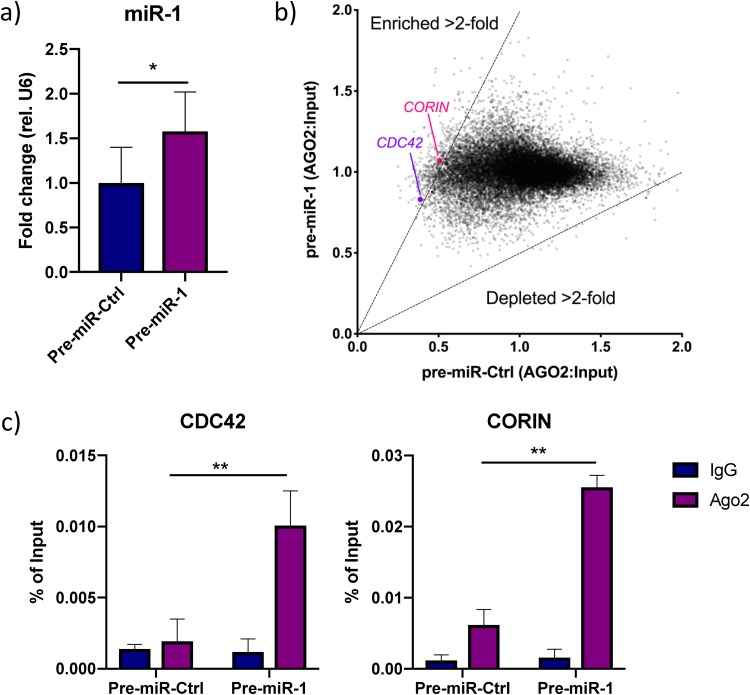
Analysis of the miR-1-3p targetome. (a) miR-1 expression in iPS-CM transfected with pre-miR-Ctrl or pre-miR-1. Results are expressed relative to the expression of U6 RNA and normalized to the mean value for the control cells. *, *P* < 0.05, using Student’s *t* test. Data are derived from three separate experiments. (b) Scatter plot showing genes enriched or depleted >2-fold in AGO2-immunoprecipitated RNA from cells transfected with pre-miR-1 compared to the results for cells transfected with pre-miR-Ctrl. The expression value for each gene is normalized to that of its input control. (c) Enrichment of *CDC42* and *CORIN* in AGO2-immunoprecipitated RNA from iPS-CM transfected with pre-miR-1 compared to their expression levels in cells transfected with pre-miR-Ctrl was validated with qRT-PCR. Shown are relative expression levels for the negative-control samples (IgG) and the AGO2 samples, normalized to the value for input RNA. Results are derived from three separate experiments. **, *P* < 0.01, comparing the levels of AGO2-associated RNA between cells transfected with pre-miR-Ctrl and pre-miR-1 using 2-way analysis of variance (ANOVA) with Sidak’s multiple-comparison test.

We next sought to comprehensively map miR-1-3p mRNA targets in human cardiomyocytes that could explain the effect on corin activity. We performed RNA immunoprecipitation (RIP) with an argonaute 2 (AGO2) antibody in iPS-CM transfected with synthetic pre-miR-1 or negative-control pre-miRNA. Overexpression of miR-1-3p was validated with qRT-PCR (Fig. 3a). Coprecipitated RNA was analyzed with microarrays, and 367 mRNAs were enriched >2-fold in the AGO2 fraction following miR-1-3p overexpression (Fig. 3b, Table S2). These mRNAs included 5 validated miR-1-3p targets (*CDC42* [30], *FN1* [31], *PDIA3* [32], *SARS* [33], and *SOD1* [34]) and 27 predicted targets (based on results from the miRNA target prediction tool Targetscan 7.2) (Table S2) (35). Interestingly, we also identified *CORIN* itself as one of the enriched miR-1/AGO2-associated mRNAs. Given the absence of other genes with serine protease activity or genes known to be transcriptional or translational regulators of corin in the list of miR-1/AGO2-associated RNAs, we concluded that miR-1-3p affects cardiac serine protease activity directly by interacting with *CORIN* mRNA. We confirmed the enrichment of *CORIN* and *CDC42* in the AGO2 fraction of cells overexpressing miR-1-3-p using qRT-PCR (*P* < 0.01) (Fig. 3c).

### miR-1 regulates *CORIN* expression through direct binding to a site in the 3′ UTR.

**FIG 4:**
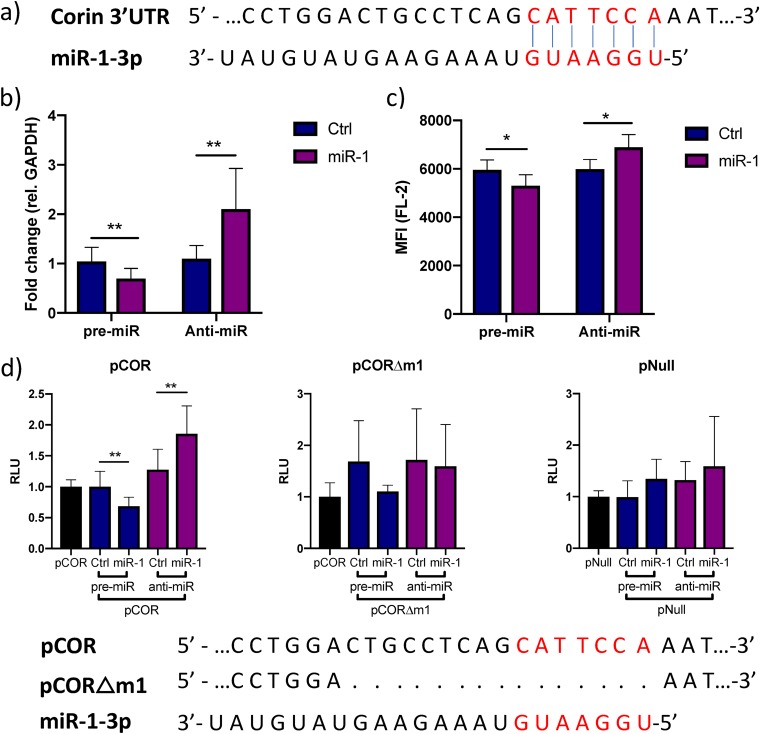
miR-1 negatively regulates corin expression through binding to a site in the 3′ UTR. (a) Representation of the predicted miR-1-3p target site (red letters) in the 3′ UTR of corin mRNA. (b) Relative expression of *CORIN* in iPS-CM transfected with pre-miR-1 or anti-miR-1 measured by qRT-PCR. Results are derived from three separate experiments. Data are normalized to the expression of *GAPDH* and expressed relative to the mean value for the respective control group. **, *P* < 0.01, using Student’s *t* test. (c) Membrane expression of corin in iPS-CM transfected with pre- or anti-miR-1 analyzed with flow cytometry. Cells were stained with a corin antibody, and the mean fluorescence intensity (MFI) in the FL-2 channel was measured. Data are derived from three separate experiments. *, *P* < 0.05 using Student’s *t* test. (d) A reporter plasmid with the 3′ UTR of corin cloned downstream from the *Gaussia* luciferase gene (pCOR) was cotransfected with pre- or anti-miR-1 (or corresponding controls) in iPS-CM, and the luminescence signal was measured after 72 h. The signal from the passive reporter gene, encoding SEAP, was used for normalization of the luciferase signal. The results are derived from three separate experiments. **, *P* < 0.01, using Student’s *t* test. The reporter assay was repeated with a plasmid in which the predicted miR-1 target site had been deleted by site-directed mutagenesis (pCORΔm1) or with an empty reporter vector (pNull).

Next, we wanted to test whether miR-1-3p regulates *CORIN* expression through direct interaction with a target site in its 3′ UTR. We identified a poorly conserved, putative 7-mer-α target site for miR-1-3p in the 3′ UTR of *CORIN* mRNA using Targetscan 7.2 (Fig. 4a). Inhibition or overexpression of miR-1-3p in iPS-CM caused significant increase or decrease in *CORIN* gene expression (Fig. 4b), as well as the amount of membrane-bound corin protein (Fig. 4c). To confirm the interaction of miR-1-3p with the predicted target site, a reporter construct carrying the *CORIN* 3′ UTR downstream from the *Gaussia* luciferase gene (denoted pCOR) was transfected into human iPS-CM. The reporter signal was significantly increased when inhibiting miR-1-3p via the LNA antisense inhibitor anti-miR-1 and decreased when overexpressing miR-1-3p (Fig. 4d), indicating that miR-1-3p interacts with the *CORIN* 3′ UTR. Deletion of the predicted target site from the reporter plasmid by site-directed mutagenesis (yielding a plasmid denoted pCORΔm1) completely abolished the ability of miR-1-3p to modulate the reporter signal, confirming the functionality of this specific sequence. Importantly, the reporter signal from the empty vector (pNull) was not affected by transfection with pre-miR-1 or anti-miR-1. These results confirm that *CORIN* is a direct target of miR-1-3p in human cardiomyocytes.

### Additional effects of miR-1 on ANP biogenesis.

**FIG 5:**
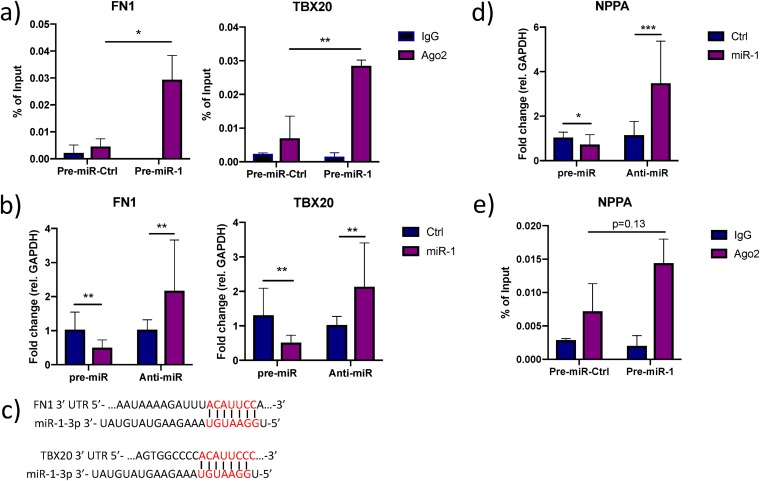
Additional effects of miR-1 on ANP biogenesis. (a) Enrichment of *FN1* and *TBX20* in AGO2-immunoprecipitated RNA from iPS-CM transfected with pre-miR-1 compared to their expression levels in cells transfected with pre-miR-Ctrl was validated with qRT-PCR. Shown are relative expression levels for the negative-control samples (IgG) and the AGO2 samples, normalized to the value for input RNA. Results are derived from three separate experiments. *, *P* < 0.05, and **, *P* < 0.01, comparing the levels of AGO2-associated RNA between cells transfected with pre-miR-Ctrl and pre-miR-1 using 2-way ANOVA with Sidak’s multiple-comparison test. (b) Relative expression of *FN1* and *TBX20* in iPS-CM transfected with pre- or anti-miR-1 measured by qRT-PCR. Results are derived from three separate experiments. Data are normalized to the expression of *GAPDH* and expressed relative to the mean value for the respective control group. **, *P* < 0.01, using Student’s *t* test. (c) Representation of the predicted miR-1-3p target sites in the 3′ UTR of ATF2, FN1, and TBX20 mRNA. (d) Relative expression of *NPPA* in iPS-CM transfected with pre- or anti-miR-1 measured with qRT-PCR. Expression data were normalized to the expression of *GAPDH* and expressed relative to the mean value for the controls. *, *P* < 0.05, and ***, *P* < 0.001, using Student’s *t* test. Results are derived from three separate experiments. (e) Level of *NPPA* in AGO2-immunoprecipitated RNA from iPS-CM transfected with pre-miR-1 compared to the level in cells transfected with pre-miR-Ctrl measured with qRT-PCR. Shown are relative expression levels for the negative-control samples (IgG) and the AGO2 samples, normalized to the value for input RNA. Results are derived from three separate experiments. Statistical significance was determined by *t* test using 2-way ANOVA with Sidak’s multiple-comparison test.

Given that a single miRNA can potentially regulate numerous target mRNAs (36) and the fact that some miRNAs have a tendency to target genes with related functions (37–40), we hypothesized that the effect of miR-1-3p inhibition on extracellular ANP levels (Fig. 2g) could be explained by other targets in addition to *CORIN* itself. To address this hypothesis, we first made a comprehensive survey of the literature and identified a total of 20 genes with well-established roles in transcriptional or translational activation of ANP expression (Table S3). Second, we cross-referenced this list with the miR-1/AGO2-associated mRNAs and identified four overlapping genes: *ATF2*, *MYOCD*, *FN1*, and *TBX20. ATF2*, *MYOCD*, and *TBX20* encode transcription factors that have been shown to activate the *NPPA* promoter (41–43), while *FN1* inhibits the binding of the repressive transcription factor NRSE to the genomic locus encompassing *NPPA* (44). Since *NPPA* is predominantly expressed in atria and the phenotype of the iPS-CM used here has been described as a mixture of atrial-, ventricular, and nodal cells (45, 46), we wanted to confirm that *NPPA* was expressed at physiologically relevant levels. qRT-PCR was performed on iPS-CM, as well as human atrial and ventricular tissue, and *NPPA* was found to be considerably more highly expressed in iPS-CM than in ventricular tissue but not as highly expressed as in atria (Fig. 5a). We validated the enrichment of *FN1* and *TBX20* in the miR-1/AGO2 fraction (Fig. 5b). Moreover, the expression of *FN1* and *TBX20* was affected by modulation of miR-1-3p in iPS-CM transfected with pre- and anti-miR-1-3p (Fig. 5c). We could also identify conserved 8-mer miRNA target sites in the 3′ UTRs of *FN1* using Targetscan 7.2 and a nonconserved 8-mer target site in the 3′ UTR of *TBX20* by manually inspecting the mRNA sequence (Fig. 5d). In line with a model where miR-1-3p targets transcriptional activators of *NPPA*, miR-1-3p inhibition and overexpression resulted in significant up- and downregulation of *NPPA* expression, respectively (Fig. 5e). We ruled out *NPPA* as a direct target of miR-1-3p based on the complete absence of target sites throughout NPPA mRNA and nonenrichment of *NPPA* in miR-1/AGO2 RNA (Fig. 5f).

Taken together, these results indicate that in addition to targeting *CORIN*, miR-1-3p influences ANP biogenesis through targeting several transcriptional activators of *NPPA* but not *NPPA* itself.

## DISCUSSION

The concept of enhancing natriuretic peptide (NP) bioavailability for therapeutic benefit has recently found its way to clinical implication. However, the current mode of NP augmentation via inhibition of neprilysin is unspecific and of limited benefit for certain HF subtypes. Thus, alternative therapeutic targets, preferably at the core of NP synthesis, could lead to improved efficacy and fewer adverse effects. The present study proposes a novel role for miR-1-3p in the regulation of ANP biogenesis. Through a systematic functional screening of miRNAs, the miR-1 family of miRNAs (including miR-1-3p and miR-206) was identified as potent regulators of corin activity in human cardiomyocytes. Mechanistically, miR-1-3p was shown to exert repressive effects by binding to a canonical miRNA binding site in the 3′ UTR of *CORIN* mRNA. Unlike previously identified transcriptional regulators of *CORIN* (4, 15), miR-1-3p expression is restricted almost exclusively to cardiac muscle (25). As such, therapeutic knockdown of miR-1-3p is less likely to have extracardiac side effects, although the benefit of such treatment must be assessed thoroughly in future studies. There is evidence that transgenic overexpression of miR-1 *in vivo* can reverse cardiac hypertrophy and remodeling (47, 48) but also promote arrhythmia (29, 49), so any attempt at modulating miR-1 therapeutically must be performed with this balance in mind. We showed that miR-1-3p is by far the predominant miR-1 family member in human cardiomyocytes and cardiac tissue, but additional effects of the other, less prevalent member (miR-206) on corin activity cannot be ruled out.

One potential benefit of using miRNAs as drug targets is that they sometimes repress multiple genes in the same pathway (50). Interestingly, we found evidence that miR-1-3p exerts additional effects on ANP biogenesis by targeting two known transcriptional activators of the *NPPA* gene, *FN1* and *TBX20*. In addition to these novel miR-1 targets, at least two previously described transcriptional activators of *NPPA* are also encoded by known miR-1 target genes: myocardin (*MYOCD*) (51) and myocardin-related transcription factor A (*MRTFA*) (52). Notably, *MYOCD* was enriched in the miR-1/AGO2 RIP fraction, thus corroborating its role as a direct miR-1-3p target in human cardiomyocytes. The fact that miR-1 seems to regulate ANP production both on the transcriptional and the posttranslational level adds further weight to it as a potent therapeutic target for NP augmentation.

## MATERIALS AND METHODS

### Cells.

Human iPS-derived cardiomyocytes (iPS-CM) were bought from Cellular Dynamics International, Madison, WI. Cells were thawed and grown in plating or maintenance medium according to the manufacturer’s instructions. All treatments were carried out at day 5 postplating. Transfections were carried out using 40 nM siRNA (Thermo Fisher) or miRNA family power inhibitor (Exiqon, Vedbaek, Denmark) and Lipofectamine 3000 (Thermo Fisher, Waltham, MA) according to the manufacturers’ instructions. RNA was prepared using the miRNeasy minikit (Qiagen, Hilden, Germany) according to the manufacturer’s instructions. The amount of ANP in the cell supernatant was determined with the atrial natriuretic peptide enzyme immunoassay (EIA) kit (Sigma-Aldrich) according to the manufacturer’s instructions.

### Tissue samples.

Human left atrial and ventricular cardiac biopsy specimens were obtained during routine clinical surveillance after transplantation or from explanted hearts. Patients provided written approval before participation, and the study was approved by the local ethics committee of Skåne University Hospital. The study was conducted in accordance with the principles of the Declaration of Helsinki.

Biopsy specimens were immediately submerged in RNAlater (Thermo Fisher) and stored at –80°C. For RNA preparation, the biopsy specimens were placed in QIAzol and homogenized with an Omni TH rotor-stator homogenizer (Omni International, Kennesaw, GA). Total RNA was prepared using the miRNeasy minikit (Qiagen) according to the manufacturer’s instructions.

### Assay for serine protease activity in cardiomyocytes.

The assay has been described previously (13) but was adapted to human cardiomyocytes. A fluorogenic bisamide derivative of rhodamine 110 (Rho110), containing two serine protease cleavage sites, was used as a substrate (Thermo Fisher). Amounts of 20,000 iPS-CM were seeded per well in black 96-well plates and left in culture for 5 days. After removal of cell medium and subsequent washing with phosphate-buffered saline (PBS), substrate diluted in 10 mM Tris buffer was added to each well and fluorescence was monitored immediately and every 5 min for 60 min using a Victor^3^ multilabel counter (Perkin Elmer, Waltham, MA). Linearity of the reaction progress curves was confirmed over a range of substrate concentrations (0.1 to 20 μM), indicating that initial reaction velocity (*V*_0_) conditions were met. *V*_max_ and *K_m_* were obtained from Michaelis-Menten saturation curves.

### miRNA family inhibitor screening.

The miRCURY LNA miRNA family power inhibitor library (Exiqon, Vedbaek, Denmark), containing inhibitors for 40 miRNA families, was used for screening. iPS-CM were seeded as described above and transfected on day 5 after plating. Cells were transfected with 40 nM miRNA family power inhibitors using Lipofectamine 3000 according to the manufacturer’s instructions. Seventy-two hours after transfection, medium was aspirated, cells washed once with PBS, and Rho110 substrate added to each well. Fluorescence was measured as described above. Two separate experiments were performed, and the *V*_0_ at 20 μM substrate was used as an endpoint in the screening. The effect of miRNA family inhibition on enzyme activity compared to that of a negative-control inhibitor was analyzed using multiple *t* tests with adjustment for multiple comparisons according to the principle of false discovery rate (*q*) (53). A *q* value of <0.05 was used as the threshold for statistical significance.

### qRT-PCR.

For analysis of miRNA expression, cDNA was synthesized using the miRCURY LNA universal reverse transcription (RT) kit and added to quantitative PCR (qPCR) mixtures containing miRCURY LNA primer sets (Exiqon) specific for miR-1-3p, miR-206, or U6 and 2× fast SYBR green master mix (Thermo Fisher).

For analysis of mRNA expression, cDNA was synthesized using the RevertAid first-strand cDNA synthesis kit with random hexamer primers and used in qPCRs with TaqMan assays specific for *CORIN*, *NPPA*, *CDC42*, *ATF2*, *FN1*, *TBX20*, and *GAPDH* (Thermo Fisher) and 2× universal TaqMan master mix (Thermo Fisher). All qPCRs were run on a StepOnePlus real-time PCR system (Thermo Fisher), and *C_T_* values were normalized first to those of their respective reference genes (*GAPDH* for mRNA and U6 RNA for miRNA expression) and second to the mean value for the control samples (ΔΔ*C_T_*) and were expressed using the 2^–ΔΔ^*^CT^* formula.

### Flow cytometry.

iPS-CM were cultured and transfected with pre- and anti-miR as described above. Seventy-two hours after transfection, 100,000 cells were trypsinized, stained with a corin antibody (1:200, MAB2209; Biotechne, Minneapolis, MN), and then washed and stained with a phycoerythrin (PE)-conjugated rat IgG antibody (1:400; R&D Systems). After washing, the mean fluorescence intensity of gated cells in the FL-2 channel was assessed using an Accuri C6 flow cytometer (BD Biosciences, Franklin Lakes, NJ). Cells stained with only secondary antibody were used as a negative control.

### AGO2-RIP.

Amounts of 1 × 10^6^ iPS-CM were seeded in 10-cm^2^ cell culture dishes. On day 5 postplating, cells were transfected with 40 nM hsa-pre-miR-1 (catalog number AM17150; Thermo Fisher) or pre-miR negative control (pre-miR-Ctrl) (catalog number AM17110; Thermo Fisher) for 72 h. RIP was performed using the Imprint RIP kit (Sigma-Aldrich) according to the manufacturer’s instructions. Cells were lysed in mild lysis buffer, including protease inhibitor cocktail, 1 M dithiothreitol (DTT), and RNase inhibitor (40 U/μl). Ten percent of the total RIP lysate was set aside as an input control. IPs were performed overnight with magnetic beads prebound with anti-Argonaute-2 antibody (5 μg, ab32381; Abcam, Cambridge, UK) or 5 μg IgG negative-control antibody. RNA was purified using phenol-chloroform extraction. TRIzol and chloroform were added to the RIP reaction mixture containing the beads, and the mixture was vortexed and centrifuged for 10 min at 16,000 × *g* at 4°C. Glycogen (5 mg/ml), 3 M sodium acetate, and 2-propanol were added to the aqueous phase and incubated for 6 h at –80°C to precipitate the RNA. After centrifugation and washing with 80% ethanol, the RNA was resuspended in nuclease-free water. Input control RNA and immunoprecipitated RNA were purified with the RNA Clean & Concentrator kit (Zymo Research, Irvine, CA) and analyzed with Human Gene 1.0 ST microarray or qRT-PCR. For microarray data, experimental quality control analyses were performed using the Expression Console software version 1.4.1.46 (Thermo Fisher Scientific). Probe summarization and data normalization were performed with the robust multiarray analysis (RMA) (54) method in the Expression Console software. Expression values were normalized to those of the input control and compared between pre-miR-1 and pre-miR-Ctrl samples. Enriched miRNAs were defined as those that were increased >2-fold in the pre-miR-1 group compared to their levels in the pre-miR-Ctrl group. qRT-PCR analysis of immunoprecipitated RNA was performed essentially as described above.

### 3′ UTR reporter assay.

For validation of the predicted miR-1 target site in the 3′ UTR of *CORIN*, an miTarget miRNA 3′ UTR target clone (Genecopoeia, Rockville, MD) was used. The expression clones were based on the pEZX-MT51 vector containing dual reporter genes, encoding *Gaussia* luciferase (GLuc) and secreted alkaline phosphatase (SEAP). SEAP serves as an internal control for normalization of transfection efficiency and cell viability. A miTarget miRNA 3′ UTR target clone containing the *CORIN* 3′ UTR (accession number NM_001278585.1) was cotransfected with 40 nM precursor miR-1 (Life Technologies), scrambled pre-miRNA, anti-miR-1 LNA antisense inhibitor, or scrambled anti-miR inhibitor into iPS-CM. Sixteen nucleotides, including the predicted miR-1 target site of the *CORIN* 3′ UTR, were deleted using the Phusion site-directed mutagenesis kit (Thermo Fisher) according to the manufacturer’s instructions, using forward primer 5′-AAT AGA TAC TAC CTG CAA TTT TAT ACA TGT-3′ and reverse primer 5′-CTG AGG CAG TCC AGG TTG-3′. Deletion of the miR-1 target sequence was confirmed with Sanger sequencing. An empty miTarget vector was transfected as a control. SEAP and GLuc activities were measured 72 h after transfection using the Secrete-Pair dual-luminescence assay kit (Genecopoeia) on a Glomax 20/20 luminometer (Promega, Madison, WI). Results were expressed as the ratio of GLuc and SEAP.

### Statistical analysis.

Data are presented as mean values and standard errors of the means (SEM). Statistical significance of differences between data for different experimental groups was assessed with *t* tests. Adjustment of *P* values for multiple comparisons was performed according to the principle of false discovery rate (53). Thresholds for statistical significance were *P* < 0.05 or *q* < 0.05. All statistical analyses and analyses of enzyme kinetics were performed in Prism version 7.00 (GraphPad, La Jolla, CA).

## Supplementary Material

Supplemental file 1
