# Supplementary material for: Functional Screening Identifies MicroRNA Regulators of Corin Activity and Atrial Natriuretic Peptide Biogenesis
Source: Mol Cell Biol. 2019 Nov 12;39(23):e00271-19. doi: 10.1128/MCB.00271-19 (PMC6851346; doi:10.1128/MCB.00271-19)
Supplement: Supplemental file 1 [file MCB.00271-19-s0001.pdf]

Supplementary Tables 1-3

Functional screening identifies microRNA regulators of CORIN activity and ANP biogenesis

Celik et al

Supplementary Table 1 – miRNA Family Inhibitor Library Contents

miRNA Family	Family members
hsa-let-7	hsa-let-7a-5p, -let-7b-5p, -let-7c-5p, -let-7d-5p, -let-7e-5p, -let-7f-5p, -let-7g-5p, -let-7i-5p, -miR-98-5p
hsa-miR-1	hsa-miR-1-3p, -miR-206
hsa-miR-10	hsa-miR-10a-5p, -miR-10b-5p
hsa-miR-103	hsa-miR-103a-3p, -miR-107
hsa-miR-125	hsa-miR-125a-5p, -miR-125b-5p
hsa-miR-130	hsa-miR-130a-3p, -miR-130b-3p, -miR-301a-3p, -miR-301b-3p
hsa-miR-132	hsa-miR-132-3p, -miR-212-3p
hsa-miR-133	hsa-miR-133a-3p, -miR-133b
hsa-miR-135	hsa-miR-135a-5p, -miR-135b-5p
hsa-miR-146	hsa-miR-146a-5p, -miR-146b-5p
hsa-miR-148	hsa-miR-148a-3p, -miR-148b-3p, -miR-152-3p
hsa-miR-15	hsa-miR-15a-5p, -miR-15b-5p, -miR-16-5p, -miR-195-5p
hsa-miR-17	hsa-miR-106a-5p, -miR-106b-5p, -miR-17-5p, -miR-20a-5p, -miR-20b-5p, -miR-93-5p
hsa-miR-18	hsa-miR-18a-5p, -miR-18b-5p
hsa-miR-181	hsa-miR-181a-5p, -miR-181b-5p, -miR-181c-5p, -miR-181d-5p
hsa-miR-19	hsa-miR-19a-3p, -miR-19b-3p
hsa-miR-190	hsa-miR-190a-5p, -miR-190b
hsa-miR-192	hsa-miR-192-5p, -miR-215-5p
hsa-miR-196	hsa-miR-196a-5p, -miR-196b-5p
hsa-miR-199	hsa-miR-199a-5p, -miR-199b-5p
hsa-miR-200	hsa-miR-141-3p, -miR-200a-3p, -miR-200b-3p, -miR-200c-3p, -miR-429
hsa-miR-204	hsa-miR-204-5p, -miR-211-5p
hsa-miR-208	hsa-miR-208a-3p, -miR-208b-3p
hsa-miR-23	hsa-miR-23a-3p, -miR-23b-3p
hsa-miR-25	hsa-miR-25-3p, -miR-92a-3p, -miR-92b-3p
hsa-miR-26	hsa-miR-26a-5p, -miR-26b-5p
hsa-miR-27	hsa-miR-27a-3p, -miR-27b-3p
hsa-miR-29	hsa-miR-29a-3p, -miR-29b-3p, -miR-29c-3p
hsa-miR-30	hsa-miR-30a-5p, -miR-30b-5p, -miR-30c-5p, -miR-30d-5p, -miR-30e-5p
hsa-miR-300	hsa-miR-300, -miR-381-3p
hsa-miR-302	hsa-miR-302a-3p, -miR-302b-3p, -miR-302c-3p, -miR-302d-3p;
hsa-302&520	hsa-miR-302a-3p, -miR-302b-3p, -miR-302c-3p, -miR-302d-3p, -miR-302e, -miR-520a-3p, -miR-520b, -miR-520c-3p, -miR-520e, -miR-520f-3p,
	hsa-miR-520g-3p, miR-520h, miR-526b
hsa-miR-320	hsa-miR-320a, -miR-320b, -miR-320c, -miR-320d, -miR-320e
hsa-miR-371	hsa-miR-371a-3p, -miR-371b-3p
hsa-miR-374	hsa-miR-374a-5p, -miR-374b-5p, -miR-374c-5p
hsa-miR-376	hsa-miR-376a-3p, -miR-376b-3p
hsa-miR-449	hsa-miR-449a, -miR-449b
hsa-miR-501	hsa-miR-501-3p, -miR-502-3p
hsa-miR-517	hsa-miR-517a-3p, -miR-517b-3p, -miR-517c-3p
hsa-miR-518	hsa-miR-518a-3p, -miR-518b, -miR-518c-3p, -miR-518d-3p, -miR-518e-3p, -miR-518f-3p
hsa-miR-519	hsa-miR-519a-3p, -miR-519b-3p, -miR-519c-3p, -miR-519e-3p
hsa-miR-520	hsa-miR-520a-3p, -miR-520b, -miR-520c-3p, -miR-520d-3p, -miR-520e, -miR-520f-3p, -miR-520g-3p, -miR-520h, -miR-526b-3p
hsa-miR-99	hsa-miR-100-5p, -miR-99a-5p, -miR-99b-5p

Supplementary Table 2 – Enriched mRNAs in AGO2-RIP

Gene Symbol	Fold enrichment	Known miR-1 target	miR-1-3p target predicted by Targetscan 7.2
AAK1	2,0		
ACSF2	2,2		
ACSL3	2,1		
ACTN2	2,0		
ADAMTSL4	2,1		
ADHFE1	2,3		
AGFG1	2,3		
AIMP2	2,1		
AK2	2,1		
ALG3	2,4		
ALG8	2,3		
ALPK2	2,2		
AP2B1	2,4		
ARG2	2,2		X
ARHGDIA	2,3		
ARHGEF9	2,1		
ARL5B	2,0		
ARV1	2,0		
ASB1	2,2		
ATF2	2,2		X
ATP13A3	2,4		
ATP1A3	2,2		
ATP1B1	2,4		
ATP5G1	2,2		
ATP5O	2,2		
ATP6AP1	2,2		
ATP6AP2	2,2		
ATP6V0B	2,1		
ATP6V0E1	2,4		
ATP6V1G2	2,0		
ATRAID	2,1		
BCL2L1	2,1		
BRD2	2,1		
BTRC	2,0		
C14orf1	2,1		
C1orf123	2,0		
C2orf42	2,5		
C8orf49	3,1		
CACHD1	2,1		
CASZ1	2,4		
CCT7	2,6		
CDC42	2,1	Qian et al 2011 J Cell Biol	X
CDK2	2,4		
CDKN1B	2,3		

Supplementary Table 2 continued.

CEP170	2,1		
CERS6	2,3		
CHP1	2,6		
CLNS1A	2,3		
CLPTM1	2,1		
CLTC	2,0		X
CMC2	2,1		
CNIH4	2,4		
CNOT1	2,1		X
CNOT7	2,2		
COA3	2,2		
COPS3	2,0		
COPZ1	2,9		
CORIN	2,1		
COX17	2,2		
COX6C	2,1		
COX7A2	2,0		
COX7B	3,2		
CRHR1	2,4		
CSTB	2,4		
CWC27	2,1		
DAD1	4,1		
DCAKD	2,0		
DCTN3	2,2		
DCTN5	2,1		
DDAH1	2,5		
DDIT3	2,0		
DHCR24	2,1		
DHCR7	2,2		
DHFR	2,6		
DIRC2	2,3		
DLST	2,0		
DTL	2,1		
DUSP16	2,4		
DYM	2,1		
DYNLT1	2,4		
ECH1	2,0		
EIF1AD	2,1		X
EIF2B3	2,3		
EIF2S2	2,3		
EIF2S3	2,2		
EIF3L	2,5		
EIF4A1	2,0		
EIF4B	2,6		
EIF5AL1	2,6		

Supplementary Table 2 continued.

ELAC2	2,3		
FADS2	2,0		
FAM118B	2,2		
FAM127B	2,1		
FAM96B	2,2		
FBL	2,4		
FERMT2	2,0		
FIGN	2,4		
FN1	2,1	Wang et al 2011 FEBS Lett	X
FXYD6	2,3		
GABARAPL1	2,8		
GABBR1	2,7		
GLB1	2,1		
GNB1	2,1		
GNS	2,1		
GOSR2	2,4		
GPRIN3	2,0		X
H3F3A	2,4		
HAX1	2,0		
HELZ	2,0		
HIBADH	2,2		
HIPK1	3,1		
HIST1H2AC	2,1		
HIST1H3B	2,2		
HIST2H2AC	2,9		
HNRNPA1	2,0		X
HNRNPK	2,2		X
HPS5	2,4		
HSD17B8	2,1		
HSP90B1	2,1		X
HSPB3	2,3		X
ILF2	2,4		
INTS7	2,0		
ITFG1	2,2		
JAK1	2,1		
JTB	2,2		
KARS	2,1		
KDELR2	2,2		X
KIAA0319L	2,1		
KLHDC2	2,0		
KPNA2	2,3		
KPNB1	2,0		
LAPTM4A	2,4		
LDOC1	2,0		
LOC728323	2,3		

Supplementary Table 2 continued.

LPCAT3	2,4		
LPIN1	2,1		
MAFG	2,3		
MAPK9	2,2		
MAPKAP1	2,2		
MARCH7	2,1		
MED13	2,2		
MED24	2,1		
MED27	2,1		
MINPP1	2,3		
MMADHC	2,5		
MRPL15	2,2		
MRPL45	2,1		
MRPL51	2,4		
MRPL53	2,1		
MRPS18A	2,0		
MRPS21	2,0		
MSN	2,1		
MT2A	2,1		
MYL12B	2,8		
MYOCD	2,8		X
NAA40	2,1		
NACA	2,3		
NCSTN	2,5		
NDUFA1	2,3		
NDUFA12	2,0		
NDUFA2	2,7		
NDUFA4	2,6		
NDUFA9	2,2		
NDUFAB1	2,1		
NDUFAF4	2,5		
NDUFB4	2,5		
NDUFB4	2,5		
NDUFS5	3,1		
NECAP1	2,2		
NFE2L1	2,0		
NGRN	2,1		
NLK	3,0		
NNT	2,2		
NOP10	2,1		
NPIPB5	3,0		
NPPB	2,1		
NSA2	2,4		
NUP214	2,2		
OGDH	2,4		

Supplementary Table 2 continued.

OR7E26P	2,4		
OSTC	3,2		
P2RX1	2,1		
PAK1	2,5		
PDIA3	2,2	Herrera-Uribe et al 2018 Vet Res	
PDK2	2,2		
PDPR	2,2		
PEBP1	2,5		
PERP	2,6		
PFN2	2,7		X
PHB	2,2		
PHF12	2,4		
PIGU	2,4		
PIP5K1A	2,6		
PLAGL2	2,2		
PLEKHA3	2,1		
PLEKHM3	3,4		
PLPP1	2,0		
PMP22	3,0		
POLR2G	2,4		
POLR2H	2,0		
POLR3C	2,3		
POM121	3,1		
POMGNT1	2,0		
POPDC2	2,2		
PPIAL4F	2,0		
PPIP5K1	2,5		
PPP1CC	2,1		
PRDX3	2,1		
PRELID3B	2,4		
PRKACA	2,1		
PRKAG1	2,0		
PRMT5	2,1		
PROS2P	2,0		
PROSER1	2,2		
PRRX1	2,1		
PSMA5	2,0		
PSMA6	2,1		
PSMB4	2,2		
PSMC2	2,2		
PSMD10	2,6		
PSMD8	3,0		
PTP4A2	2,5		
PTP4A3	2,1		
PTPRM	2,1		

Supplementary Table 2 continued.

QARS	2,0		
RAB2A	2,1		
RAB7A	2,4		
RASSF4	2,0		
RBBP7	2,2		
RBM14	2,1		
REEP3	2,3		
REEP5	2,4		
REPS1	2,0		
RHOBTB3	2,1		
RNF115	2,0		
RPA2	2,0		
RPL10	3,6		
RPL13	2,3		
RPL15	2,3		
RPL24	2,1		
RPL29	2,1		
RPRD2	2,0		
RPS13	3,3		
RPS2P5	2,6		
RPS3A	2,2		
RTF1	2,1		
RYBP	2,1		X
SARS	2,0	Lin et al 2013 Nat Commun	
SCD	2,3		X
SCUBE3	2,6		
SDF2	2,2		
SDHAF2	2,3		
SDHB	2,0		
SEC61A1	2,3		
SEC63	2,0		X
SERINC3	2,1		
SF3B1	2,0		
SHISA5	2,1		
SIDT2	2,1		
SIRPA	2,4		
SLC25A37	2,3		
SLC31A1	2,1		X
SLC35A4	2,2		
SLC38A1	2,5		
SLC38A2	2,5		X
SLC38A3	2,1		
SLC44A2	2,1		X
SLC4A1AP	2,5		
SLC7A1	3,2		

Supplementary Table 2 continued.

SLIRP	2,6		
SMDT1	2,1		
SMG1P1	2,8		
SMG1P5	2,7		
SMG7	2,2		X
SMIM19	2,1		
SMIM4	2,2		
SNHG17	2,8		
SNORA23	2,5		
SNORA50A	2,2		
SNORD59B	2,3		
SNU13	2,1		
SOD1	2,1	Wang et al 2015 Cell Stress Chaperones	
SPPL3	2,1		
SPRY4	2,0		
SPRYD4	2,3		
SREBF2	2,0		
SRSF7	2,1		
SSR2	2,5		
STAT2	2,1		
STMN1	2,8		
STT3A	2,2		
SUPV3L1	2,3		
TAF9B	2,0		
TANC2	2,7		
TBCK	2,2		
TBX20	2,2		
TEAD1	2,9		
TECRL	2,2		
TFG	2,1		
TM9SF2	2,2		
TM9SF4	2,1		
TMCO1	2,2		
TMED2	2,1		
TMED9	2,1		
TMEM115	2,4		
TMEM116	2,3		
TMEM126A	2,1		
TMEM138	3,1		
TMEM141	2,1		
TMEM14C	2,2		
TMEM167A	2,2		
TMEM183A	2,4		
TMEM184B	3,4		
TMEM203	2,5		

Supplementary Table 2 continued.

TMEM218	3,0		
TMEM230	2,4		
TMEM47	3,1		
TMEM59	2,2		
TMEM63B	2,1		
TMLHE	2,0		
TMX1	2,3		X
TOMM7	2,3		
TOX	2,1		
TOX4	2,0		
TPGS2	2,0		
TRAM2	2,1		
TRAPPC1	2,6		
TRAPPC4	2,3		
TRMT112	2,8		
TSFM	2,6		
TSPAN18	3,6		
TSPAN6	2,2		
TUBG1	2,2		
TUG1	3,0		
TXN	2,1		
TXNDC12	2,0		
UBE2E2	2,2		
UBE2F	2,5		
UBE2T	2,8		
UBE4B	2,1		
UCHL1	2,5		
UGDH	2,2		
UGP2	2,2		
UQCR10	2,5		
UQCR11	2,0		
UQCRH	2,4		
USMG5P1	2,7		
USP2	2,2		
USP4	2,1		
UXT	2,1		
VMP1	2,2		X
VPS39	2,6		
VTI1B	2,9		
WBP2	2,2		X
WDR6	2,1		X
WDTC1	2,0		
WIPI1	2,1		
WLS	2,3		
WNK1	2,1		

Supplementary Table 2 continued.

WSB1	2,9		
YBX3	2,4		
YIF1A	2,1		
ZDHHC9	2,3		
ZNF106	2,2		
ZNF778	3,4		
ZNFX1	2,0		
ZYX	2,2		

Supplementary Table 3 – List of genes involved in ANP biogenesis

Gene Symbol	Regulatory mechanism	References
SRF	Transcriptional activator of NPPA	Sprenkle et al, Circ Res 1995 Dec;77(6):1060-9
		Thuerauf et al J Biol Chem 1998 Aug;273(32):20636-43
ATF2	Transcriptional activator of NPPA	Lim et al, J Mol Cell Cardiol 2005 Oct;39(4):627-36
ATF6	Transcriptional activator of NPPA	Thuerauf et al J Biol Chem 1998 Aug;273(32):20636-43
NKX2-5	Transcriptional activator of NPPA	Lee et al, Mol Cell Biol 1998 Jun;18(6):3120-9
		Durocher et al, Mol Cell Biol 1996 Sep; 16(9):4648-55
		Shiojima et al, J Biol Chem 1999 Mar 19;274(12):8231-9
GATA4	Transcriptional activator of NPPA	Durocher & Nemer, Dev Genet 1998;22(3):250-62
GATA6	Transcriptional activator of NPPA	Temsah & Nemer, Regul Pept 2005 Jun 30;128(3):177-85
TBX5	Transcriptional activator of NPPA	Bruneau et al, Cell 2001 sep 21;106(6):709-21
		Hiroi et al, Nat Genet 2001 Jul;28(3):276-80
TBX20	Transcriptional activator of NPPA	Stennard et al, Dev Biol 2003 Oct 15;262(2):206-24
MEF2C	Transcriptional activator of NPPA	Morin et al, EMBO J 2000 May 2;19(9):2046-55
BAF60C	Transcriptional activator of NPPA	Lickert et al, Nature 2004 Nov 4;432(7013):107-12
PITX2	Transcriptional activator of NPPA	Ganga et al, J Biol Chem 2003 Jun 20;278(25):22437-45
HAND2	Transcriptional activator of NPPA	Thattaliyath et al, J Mol Cell Cardiol 2002 Oct;34(10):1335-44
FOS	Transcriptional activator of NPPA	Rosenzweig et al, Circ 1991 Sep;84(3):1256-65
JUN	Transcriptional activator of NPPA	Rosenzweig et al, Circ 1991 Sep;84(3):1256-65
NR3C1	Transcriptional activator of NPPA	Argentin et al, J Biol Chem 1991 Dec 5;266(34):23315-22
FN1	Transcriptional activator of NPPA	Ogawa et al, Cardiovasc Res 2002 Feb 1;53(2):451-9
MYOCD	Transcriptional activator of NPPA	van Tuyn et al, Cardiovasc Res 2005 Aug 1;67(2):245-55
MTRFA	Transcriptional activator of NPPA	Hinson et al, Am J Physiol Heart Circ Physiol 2008 Apr;294(4):H1939-47
NPPA-AS1	Post-transcriptional regulation of NPPA	Annilo et al, BMC Mol Biol 2009 Aug 11;10:81
CORIN	Post-translational processing of ANP	Yan et al, Proc Natl Acad Sci U S A 2000 jul 18;97(15):8525-9
